# Allogeneic hematopoietic stem cell transplantation for relapsed acute myeloid leukemia in ETO positive with reduced-intensity conditioning

**DOI:** 10.18632/oncotarget.22612

**Published:** 2017-11-03

**Authors:** Zhi Guo, Chen Xu, Hu Chen

**Affiliations:** ^1^ Department of Medical Oncology, National Cancer Center/Cancer Hospital and Shenzhen Hospital, Chinese Academy of Medical Sciences and Peking Union Medical College, Shenzhen, 518116, China; ^2^ Center of Hematopoietic Stem Cell Transplantation, 307 Hospital of People's Liberation Army, Beijing, 100071, China

**Keywords:** reduce intensity, allogeneic hematopoietic stem cell transplantation, ETO positive, acute myeloid leukemia, relapsed

## Abstract

**Objective:**

This research is conducted under the intention of exploring the efficacy and safety of reduced-intensity conditioning for allogeneic hematopoietic stem cell transplantation (allo-HSCT) in the treatment of relapsed ETO positive acute myeloid leukemia (AML).

**Materials and Methods:**

Treatment of 15 cases referring to recurrent ETO positive acute myeloid leukemia in an army hospital from January 2010 to January 2013 through allo-HSCT with reduced-intensity conditioning. All participants belonged to the recurrent or refractory type, including 10 males and 5 females, aging from 16 to 48 years old, with the average age of 32.5 years old. Before transplantation, 6 cases were remission while 9 were not, 10 cases were HLA-identical matching and 5 cases were HLA-haploidentical. Donors received G-CSF to mobilize and used peripheral blood stem cell transplantation. Patients received a combination of Fludarabine, Busulfex and cytarabine as conditioning regimen. Preventive donor peripheral blood stem cell infusion was used 3 months after transplantation in order to observe toxicity, graft versus host disease(GVHD) and disease-free survival.

**Results:**

All patients reached hematopoietic reconstitution, the average time were 15.5d and 16.8d respectively with neutrophils > 0.5 × 10^9^/L and platelets > 20 × 10^9^/L. Engraftment was confirmed by the evidence of 100% donor hematopoiesis and T lymphocyte subsets counts increased significantly before and after transplantation. Univariate analysis showed that the levels of CD3+, CD4+, CD8+, CD19+ significantly increased after transplantation (*P* < 0.05) . Until June 2016 after the duration of 27.5 months, 8 cases presented the presence of GVHD, one died of complication, another 4 died of relapse and the other three remained disease-free survival, the DFS rate of 2-year was 66.7%, with the longest DFS up to 54 months. Considering of the transplantation cases with remission into relief groups (6 cases), and not ease group (9 cases), 2 years of disease-free survival rates were 66.7% and 66.7%. The survival curves of the two groups are demonstrated with no significant statistical significance (*P* > 0.05).

**Conclusions:**

Reduced-intensity allogeneic hematopoietic stem cell transplantation remains effective for relapsed AML with ETO positive, with safe and effective features and can be used as the method for relapsed AML with ETO positive.

## INTRODUCTION

ETO (Eight-Twenty-One, also called myeloid translocation gene on 8) fusion gene is regarded as the most frequent abnormality observed among approximately 45% of patients with acute myeloid leukemia (AML) with French-America-British-M2 morphology and an aneuploid karyotype, in which a reciprocal translocation, t (8; 21)(q22; q22) t(8; 21), brings together a large proportion of ETO gene from chromosome 8 and part of the AML1 gene from chromosome 21 to form AML1/ETO fusion gene. It’s considered to be associated with relatively favourable prognosis. ETO fusion gene positive AML patients demonstrate a better response rate and long-term survival rate. Considering of those ETO fusion gene positive patients with high white blood cell number at first diagnosis, bone marrow involvement, addition chromosomes, expression of CD56 or expression of c-kit gene are at high risk. These high-risk patients are confronted with poor prognosis and extremely high recurrence rate [1–2]. The majority of these high-risk patients will eventually die from complications of chemotherapy, recurrence or progression. Allogeneic hematopoietic stem cell transplantation(allo-HSCT) is the best choice for these high-risk patients [3–4]. However, most patients have been treated with intensive chemotherapy for many times before the recurrence, especially for combination regimen based on high dose cytarabine and the relapsed and refractory leukemia patients normally have difficulty in accepting the allogeneic hematopoietic stem cell transplantation. Once, reduced intensity pretreatment allo-HSCT for AML1/ETO positive patients was carried out. At present stage, diagnosis and treatment works of 15 cases concerning relapse AML in ETO positive patients are summarized in this paper.

## RESULTS

**Figure 1:**
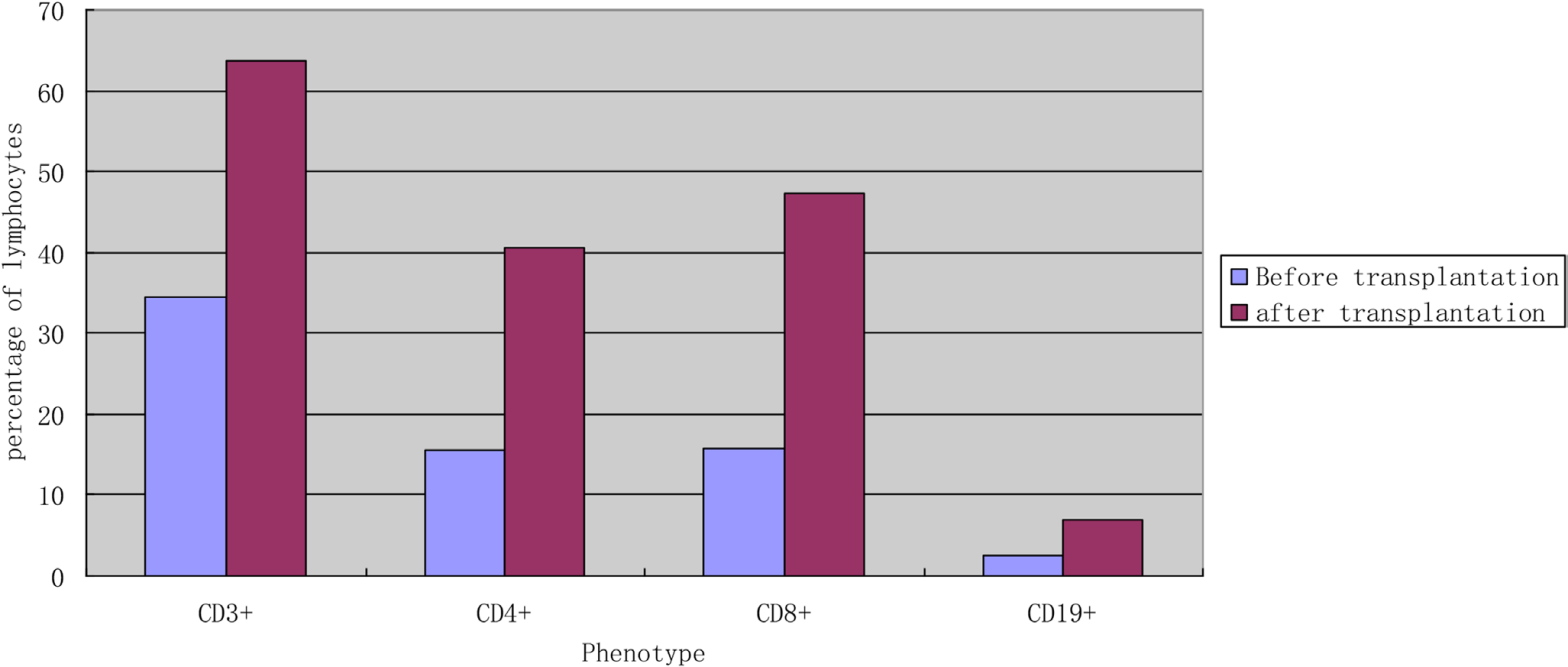
The average percentage of subtype lymphocytes according to cell surface markers in the peripheral blood before and after transplantation (*n* = 15) Remarks: univariate analysis showed that the levels of CD3+, CD4+, CD8+, CD19+ significantly increased after transplantation(*P* < 0.05).

The median number of inputted mononuclear cells was 13.1 (5.1∼22.5) × 10^8^/kg, while median number of CD34+ cells was 4.5 (3.1∼5.9) × 10^6^/kg. All patients gained hematopoiesis reconstruction after transplantation. The time while neutrophils > 0.5 × 10^9^/L was 12∼20 days, and the average time was 15.5 days. The time while platelet > 20 × 10^9^/L was 14∼22 days, and the average time was 16.8 days. Conclusion from implant analysis indicates that donors hematopoietic were completely 100% on +30 day, +60 day, +90 day after transplantation. Immune function in all patients recovered significantly and remarkably increased the proportion of immune cells along with monitor of the level of T-lymphocyte subsets before and after transplantation. Generally speaking, all patients can tolerate preprocessing scheme better, with only mild toxic reaction of pretreatment, such as digestive tract reaction, anaphylaxis and fever reaction. These reactions disappeared after symptomatic treatment, with no serious complications of transplantation occurring afterwards, such as hepatic vein occlusion syndrome, lung infection, septicemia, viscera function failure, septic shock, cerebral hemorrhage (see Tables 1, 2; Figure 1).

**Table 1:** Outcome of patients after allo-HSCT

No	MNC (× 10^8^/kg)	CD34^+^ cell (× 10^6^/kg)	Time of ANC > 0.5 × 10^9^/L (d)	Time of PLT > 20 × 10^9^/L (d)	Outcome
1	8.4	3.9	15	20	Alive
2	8.6	4.8	20	18	Dead
3	11.2	3.6	19	16	Alive
4	13.3	3.9	12	14	Alive
5	18.5	5.3	14	22	Alive
6	14.6	4.3	13	17	Dead
7	5.1	3.2	20	15	Dead
8	9.9	3.6	20	17	Alive
9	22.5	5.3	16	18	Dead
10	12.5	5.1	12	16	Alive
11	14.6	5.9	15	17	Alive
12	6.4	4.1	13	16	Dead
13	11.1	4.3	13	15	Alive
14	21.3	5.2	16	17	Alive
15	17.5	5.0	14	15	Alive

**Table 2:** T-lymphocyte subsetsbefore and aftertransplantation of the whole group

No	CD3^+^			CD4^+^		CD8^+^		CD19^+^	
				(Before transplantation, after transplantation) (%)						
1	31.2	50.3		8.9	34.3	7.2	45.5	1.8	5.1
2	41.3	63.6		18.7	44.1	13.8	48.5	1.7	7.1
3	23.4	73.8		16.9	54.2	11.2	55.3	2.5	8.2
4	35.4	53.2		18.7	46.7	13.8	49.5	3.5	6.5
5	37.3	63.1		8.9	53.7	5.6	41.2	1.6	6.1
6	44.5	62.5		16.8	46.1	16.2	68.7	1.8	7.3
7	33.5	76.2		15.6	52.6	16.7	45.6	3.4	5.6
8	21.9	80.2		13.5	47.3	15.7	47.8	4.2	7.3
9	51.4	65.3		8.4	44.5	4.6	25.5	1.4	6.1
10	34.3	53.2		14.5	26.7	12.5	35.6	1.6	5.6
11	33.4	53.7		14.2	40.3	16.7	45.5	2.9	7.8
12	23.5	67.4		15.3	39.7	15.2	39.6	2.5	7.5
13	27.5	74.3		21.4	38.7	18.6	57.3	2.6	8.6
14	45.1	52.6		26.5	45.3	26.3	48.2	2.8	6.8
15	34.2	66.3		15.4	36.9	41.2	55.6	3.4	7.6
Mean	34.5	63.7		15.6	40.5	15.7	47.3	2.5	6.9

### Complications after transplantation

A total of 8 cases of GVHD appeared in all patients, among which 5 with acute GVHD. The median time to aggregation of acute GVHD was 43.6 days(the scope was 31∼76days). The cumulative incidence was 33.3%, according to different degree of seriousness, all cases can be divided into several conditions, 3 cases belong to II degree of GVHD∼1 case to III degree and 1 case to IV degree. 2 cases of acute GVHD were HLA-identical matching, and another 3 cases were HLA-haploidentical. In addition to the conventional drugs used for preventing GVHD, HLA-haploidentical patients had all adopted ATG during pretreatment. If GVHD occurred, GVHD resistant drug therapy was chosen according to severity degree. Intestinal occurred in 2 cases and 3 cases of liver according to the classification with parts. 3 patients could be evaluated as the patients of chronic GVHD. Among them, 1 case was HLA-identical matching and another 2 cases were HLA-haploidentical. The median time to chronic GVHD was 180 days (the scope was 120∼420days), and the cumulative incidence was 22%. 1 case was limitation GVHD, mainly associated with disorder in mouth, skin and eye. The other 2 cases were chronic extensive GVHD, most commonly happened on skin. A total of 1 case GVHD have died, the patient with IV degree of intestine GVHD and haploidentical transplantation, applied various anti GVHD treatment, including methylprednisolone, cyclosporine, mycophenolate mofetil, tacrolimus, rituximab, anti thymocyte globulin, anti-CD25, infliximab, mesenchymal stem cells, but all of these treatment is invalid, and finally died of GVHD in half year or so after transplantation. After transplantation total pulmonary infection appeared in 5 cases, CMV disease in 3 cases and 2 cases of hemorrhagic cystitis.

### ETO monitoring

The Monitoring ETO fusion gene ratio was carried out on all patients, among which 7 cases (46.7%) indicates that ETO quantitative testing is 0 one month after first transplantation then ETO quantitative are 0. There are 2 cases (13.3%), which the ETO fusion gene ration have not reached 0 one month after transplantation (the median value of 0.016%, and 0.012%∼0.018%), without intervention and close monitoring of cases, the ETO quantitative gradually fell to 0 three months after transplantation and follow-up period continuously monitor ETO quantitative were 0. There are 2 cases (13.3%) patients with ETO fusion gene ration level on the high side. Through regular monitoring of ETO fusion gene ration, it still does not turn to negative at the time of the last follow-up, the quantitative fusion gene remains at 10–6 levels, and the increase has not been higher than 1 logarithmic. The cell genetics or recurrence of hematology does not appear during follow-up. Other 4 cases (26.7%) demonstrate higher ETO fusion gene quantitative level one month after transplantation, and regular monitoring of ETO fusion gene gradually increase. Although we reduce/stop immune inhibitors and donor lymphocyte infusion, they finally died of leukemia relapse.

### Method of ETO gene testing

**Figure 2:**
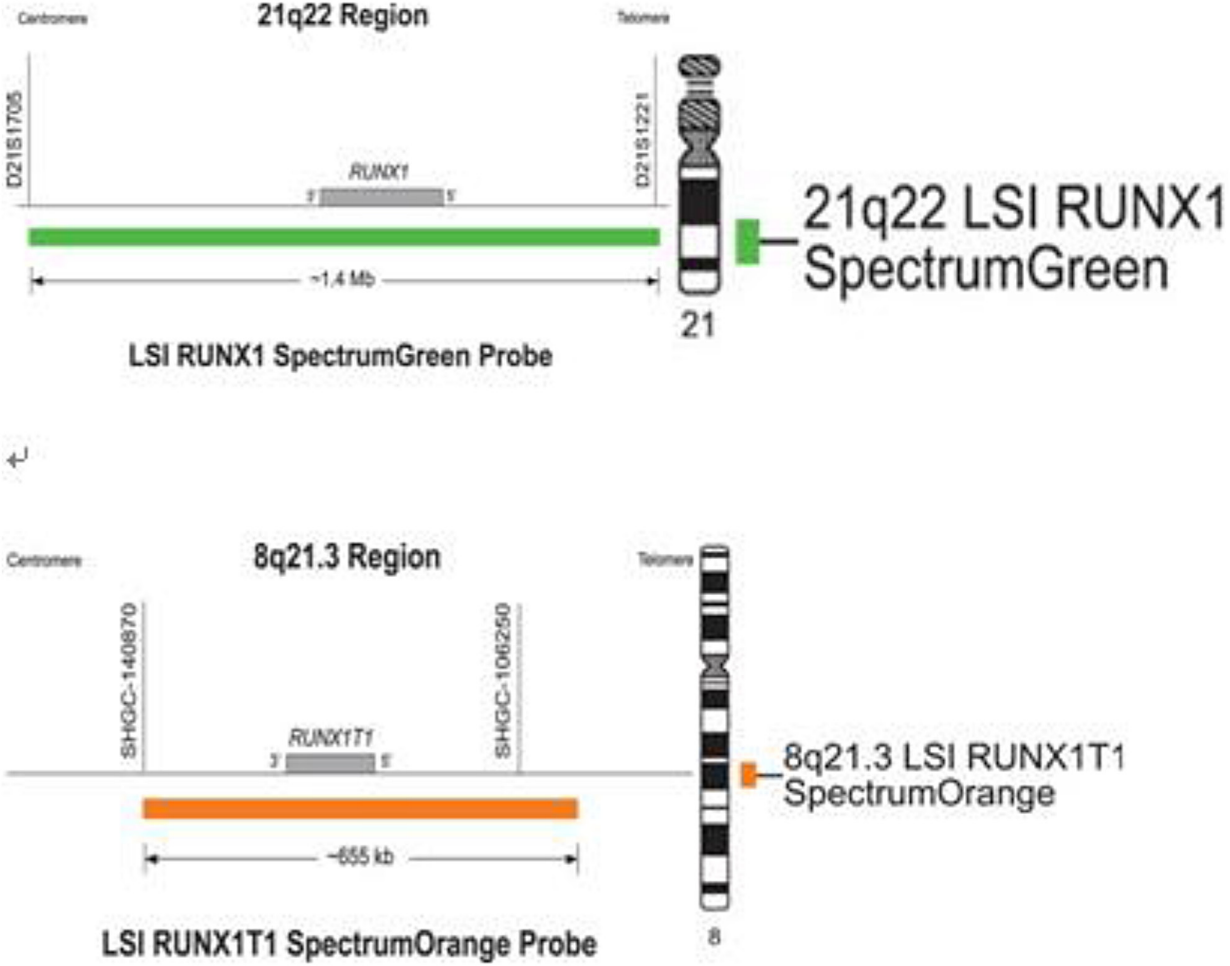
The probe map used for ETO genes

**Figure 3:**
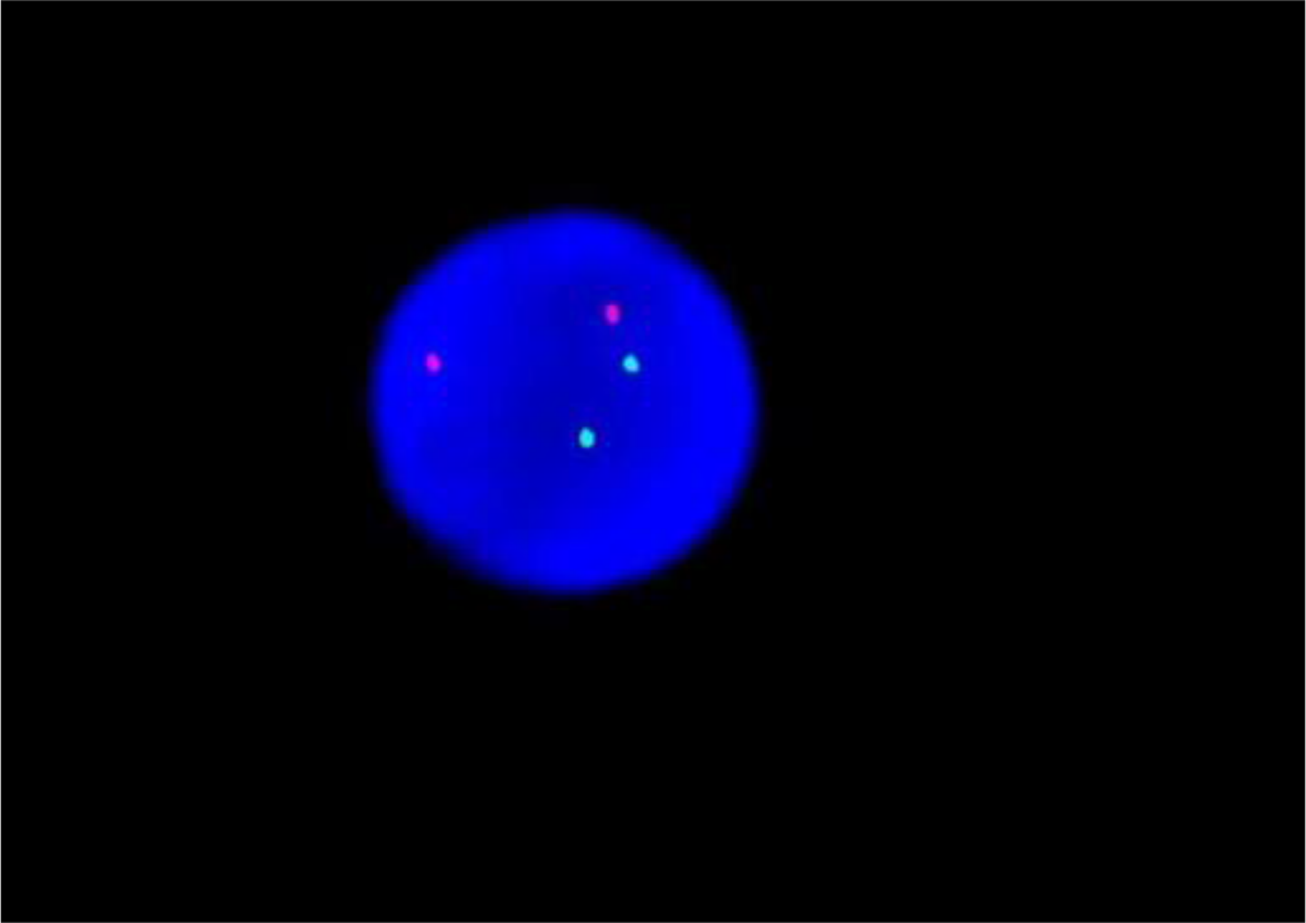
The normal ETO diagram of PCR testing

**Figure 4:**
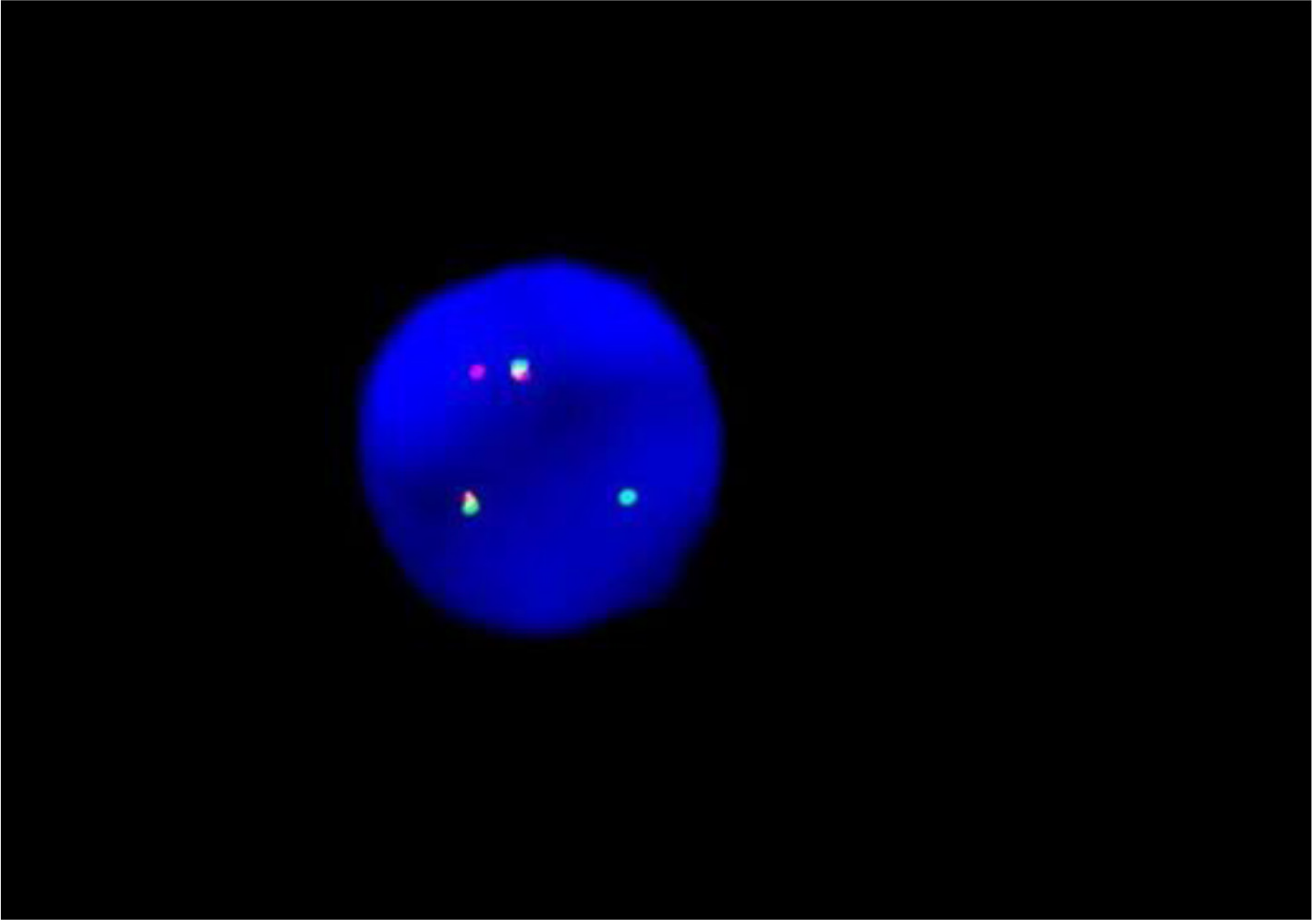
The ETO fusion diagram of PCR testing

By fluorogenic quantitative PCR, centrifugation of sampled bone marrow or blood, which ACK Lysis Buffer had been added into, was conducted. Then, DNAs were extracted to be amplified. After adding samples according to the amplification system, each amplification sequence was operated in corresponding reaction conditions. The probe used for ETO genes is: Vysis LSI RUNX1/RUNXITI Dual Color, Dual Fusion Translocation Probe (Model: 08L70–020), probe map (see Figure 2). Metasystems ISIS software was applied to signal analysis. The principle of cell counting is: within a nucleus, if the distance between two signals is less than the diameter of one signal with obviously visible yellow by superposition of red and green, they can be regarded as fusion signals. Points for attention: ①. Selected cell nuclei should be complete with a clear boundary and a proper size and without fragmentation or overlapping. ②. Signals of selected cell nuclei should be bright and analysis of cells with excessively dispersed signals should be avoided. ③. By regular movement of lens (up, down, left and right), each analyzable cell should be analyzed without arbitrary omission. ④. Signal patterns of nuclei should be recorded and proportions of cells of each signal pattern to total cells should be figured out. ⑤. Detection results should be evaluated according to CUTOFF value of each positive signal pattern established by this laboratory. ⑥. After analysis of 200 cell nuclei, when the outcome is close to CUTOFF value, counting cells should be increased to 500. The normal ETO diagram (see Figure 3) and the ETO fusion diagram (see Figure 4) by fluorogenic quantitative PCR.

### Outcome of transplantation

**Figure 5:**
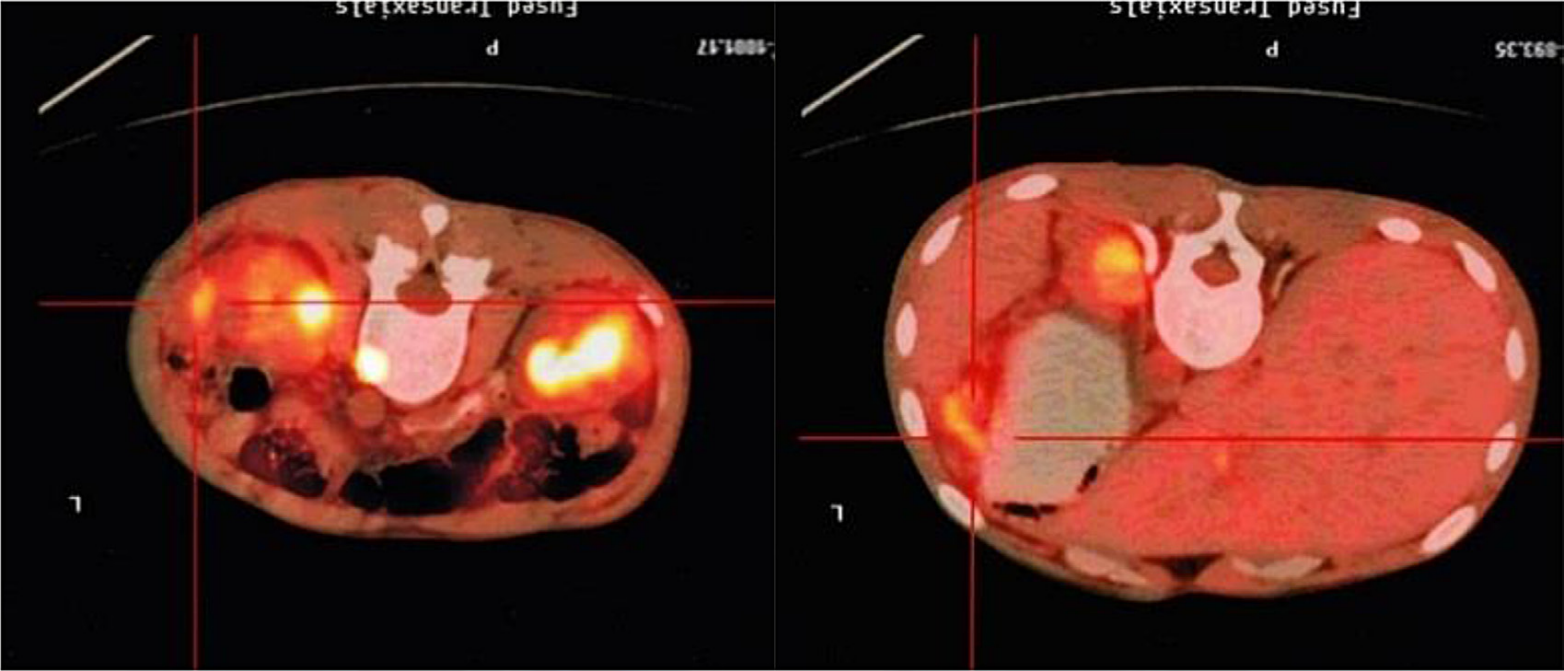
PET-CT image of a patient (male, ETO+, AML) showing widespread violations of granulocytic sarcoma

**Figure 6:**
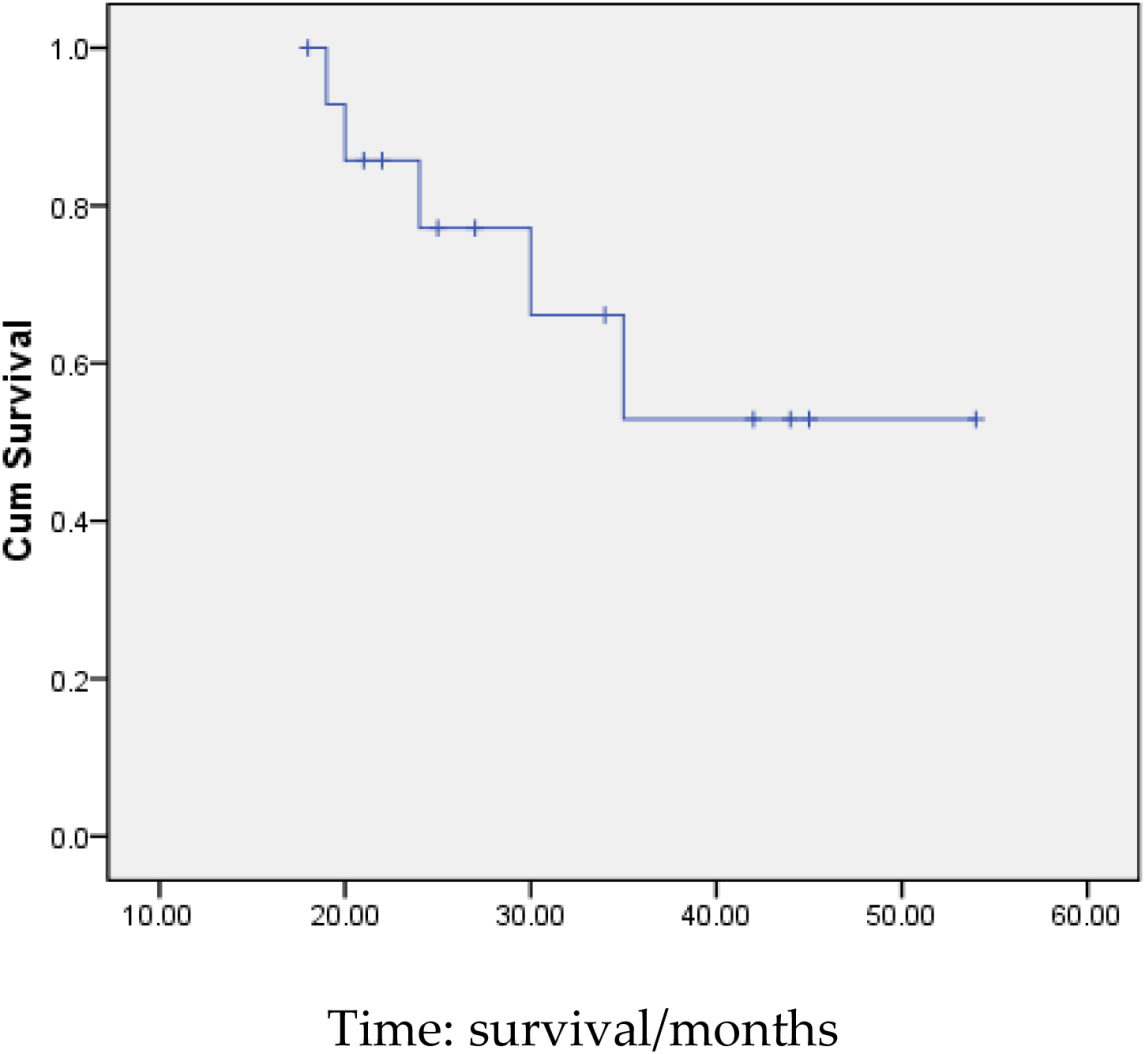
Survival curves of all patients

**Figure 7:**
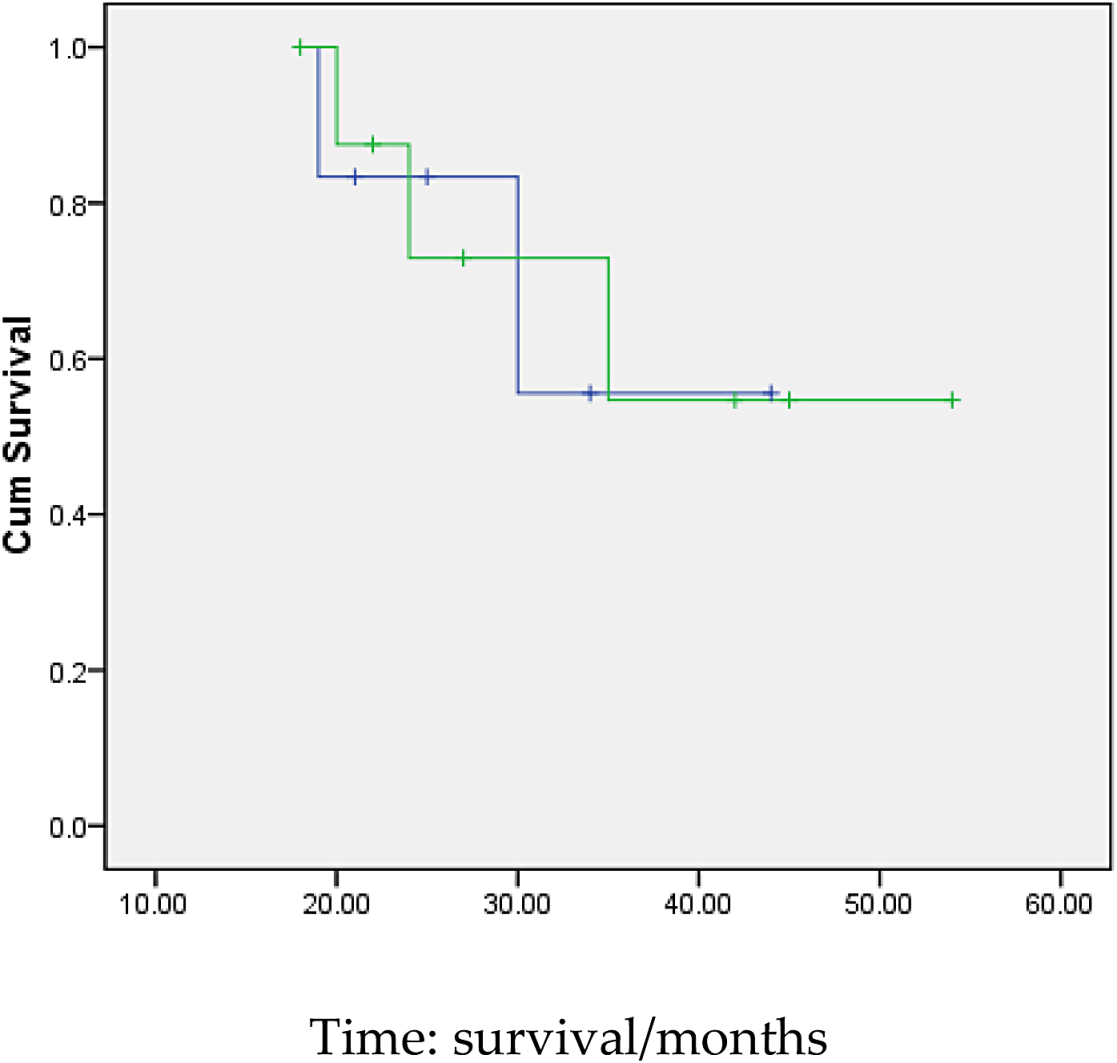
Survival curves of the two groups of patients(Remission and non-remission group) Remarks:the survival curves of the two groups are equipped with no significant statistical significance(*P* > 0.05).

3 months after transplantation of preventive donor peripheral blood stem cells, 1 bag donor peripheral blood stem cells are infused and the mononuclear cell count reaches 0.2 × 10^8^/kg. If disease recurrence appears or ETO fusion gene level increases, all the rests of the donor peripheral blood stem cells are infused. Then all patients show no symptom of GVHD or that of other complications. All patients are carried out with a follow-up visit after the transplantation, and the duration till the visit remains 27.5 months (18∼54 months), the follow-up visit lasts till June, 2014. Under statistical survival situation, all cases occurred GVHD, 1 case of death due to complications, 4 cases of death due to relapse, the rest of the 10 cases are still disease-free survival (DFS), 2 years of disease-free survival rate was 66.7%, the longest disease-free survival time was up to 54 months. In accordance with the transplantation of patients with remission into relief groups (6 cases), and not ease group (9 cases), 2 years of disease-free survival rates were 66.7% and 66.7%. The survival curves of the two groups are equipped with no significant statistical significance(*P* > 0.05).There is 1 case of 4 months after transplantation in patients with medullary recurrence, infringement of eyes, shoulder and femur, line PET-CT examination more than in the whole body metabolism active mass lesion, consider to marrow granulocyte sarcoma, before mass located in the mediastinum, the lung, stomach, spleen, kidney and left along the greater curvature rectum, large green tumor cells, blood visible last multiple organ failure caused by outside pulp widespread violations of death as shown in Figure 5, survival curve as shown in Figures 6, and 7.

## DISCUSSION

The research progress of the ETO fusion gene in the molecular genetics is rapid [5–7]. The remission rate of induction chemotherapy in patients remains high, which has been considered as one of the best prognosis subtypes in clinic [8]. After consolidation chemotherapy, long-term survival can be achieved, especially for large dose cytarabine consolidation chemotherapy with more obvious advantages and oriented just for the dangerous patients. High-risk patient prognosis, especially in conjunction with the onset of pulp outside invasion, CD56 expression, additional chromosome or c-kit gene expression, is conducted for patients who show rare cases of combined pulp outside invasion in the beginning, and there will be early intervention after hematopoietic stem cell transplantation, additional chromosome are relatively common, under the intention of affecting the prognosis of patients, which builds up the important factors for long-term survival of patients [9–10]. The c-kit gene is a proto-oncogene, and c-kit mutation is closely related to the ETO fusion gene positive AML. It is a coded transmembrane receptor tyrosine protein kinase and the receptor ligands is stem cell factors which play an important role in regulating in proliferation and differentiation of normal hematopoietic cells and tumor cells proliferation and malignant progression and so on. The c-kit gene mutations are caused by ligand stem cell factor receptor activation, characterized by excessive cellular proliferation and apoptosis resistance [11–12]. By interfering with normal hematopoietic cell proliferation, differentiation and apoptosis, it eventually leads to the occurrence of leukemia. ETO fusion gene in AML patients with positive expression c-kit gene mutation rate is 10.5%∼48.1%. The recurrence rate after these patients relieve remains high and the prognosis is poor, which should be performed allogeneic hematopoietic stem cell transplantation as soon as possible [13–15].

ETO fusion gene positive patients need not only monitoring of ETO quantitative level, but also regular detection of c-kit mutation in order to guide clinical treatment [16–18]. Literatures report the important influence on immune phenotype and cytogenetics for ETO fusion gene in AML [19], the two have already been important and independent factors. The CD56^+^ the non-myeloid antigen frequency will be significantly higher among patients with ETO positive expression [20–21], but these patients with myeloid peroxidase remain positive, CD79 A and other B cell marks are negative. But the expression of CD56^+^ does not refer to the index for the ETO fusion gene positive of patients with AML. It is suggested by the literature that it would be accompanied by its expression and easy to relapse, prognosis for high-risk independent type and hematopoietic stem cell transplantation [22–23] also needs to be conducted. The ETO fusion genes are equipped with a striking feature which is good for disease condition monitoring after transplantation. For example, after transplantation the ETO fusion gene quantitative was 0 over a period of time (usually 2 years) and the recrudescent possibility is extremely low. If the ETO fusion gene had not reached 0 early or it gradually fell to 0 without intervention and close monitoring, or the monitor remained 0 during follow-up period continuously, the probability of recurrence was also low. If regular monitoring of ETO fusion gene ration was still negative at the time of the last follow-up but all around 10–6 levels rise (range does not exceed a logarithmic), the possibility of a hematology recrudescent is low, but after transplantation the monitoring ETO fusion quantitative gradually increases, in most cases, it will appear in a different time of leukemia relapse comprehensive [24–25].

The allogeneic hematopoietic stem cell transplantation is considered as the best way to cure acute myeloid leukemia relapse type [26–27], but ETO positive patients are treated with early chemotherapy repeatedly, especially for application of large dose cytarabine which generally strengthens chemotherapy, the main viscera function and bone marrow hematopoietic function also gradually decline, and mortality is higher caused by the relapse after allogeneic hematopoietic stem cell transplantation and chemotherapy process.Dose-response relationship between the pretransplant conditioning regimen and long-term outcome after allogeneic hematopoietic stem cell transplantation in AML,Allogeneic hematopoietic transplantation produces significantly higher remission rates than chemotherapy, but only a small fraction of all patients with these diseases receive a transplant, primarily because of concerns regarding the toxicity of the preparative regimen and treatment related morbidity and mortality. so it is more urgent to ensure the safety of transplantation methods and the ones with higher cure rate. It is effective to use retrospective allogeneic hematopoietic stem cell transplantation. If the standard pretreatment intensity of allogeneic hematopoietic stem cell transplantation was taken, the related transplantation mortality was high [28]. Previous data has demonstrated that the risk of relapse is associated with the intensity of the conditioning regimen.With more intensified chemoradiotherapy, relapse is decreased.More intensive conditioning regimen is associated with a reduced risk of relapse after allogeneic hematopoietic stem cell transplantation, but does not translate in improvement of survival due to increased treatment-related mortality and nonrelapse mortality.Reduced intensity conditioning transplantation through graft-versus-leukemia effect may provide previously unavailable opportunities to cure leukemia without the morbidity and mortality associated with conventional myeloablative conditioning transplantation. The balance between the increased anti-leukemia efficacy of allogeneic hematopoietic stem cell transplantation and the risk of nonrelapse mortality depends on the disease risk category and recipients’ physical fitness and source of the transplanted stem cell.

Treatment with conventional allogeneic hematopoietic cell transplantation has been limited to strong patients because high-dose cytotoxic conditioning regimens are poorly tolerated by the relapse AML in ETO positive patients.recent years have seen the introduction of various reduced-intensity and nonmyeloablative conditioning regimens for allogeneic hematopoietic stem cell transplantation .The principles of this approach included reduction of regimen-related toxicities and shifting the burden of tumor cell kill from high-dose cytotoxic therapy to graft-versus-leukemia effects. Therefore, pretreatment has been arranged to reduce the intensity of allogeneic hematopoietic stem cell transplantation, and it no longer relys on strong radiation and chemotherapy to kill leukemia cells which reduce the recurrence of pretreatment intensity of chemotherapy, so it can still aims at achieving clear tumor in patients with refractory acute myeloid leukemia [29]. Then leukemia cells would be cleared through graft versus leukemia effect after implant [30]. The graft versus leukemia effect after transplantation was quite strong, if the intensity of transplantation during pretreatment can be reduced and the implantation can go across the immune barrier to hematopoietic system reconstruction after transplantation immune system, playing a graft versus leukemia effect, it is more important to reduce the related complications of transplantation and increase the feasibility of transplantation, then the reduced intensity of pretreatment for ETO recurrent acute myeloid leukemia belongs to a kind of remedial treatment. The patients have been recurrence refractory, resistant to chemotherapy, taking effect by reducing the intensity of the pretreatment of leukemia, after stem cells infusion to implant state, through graft versus leukemia effect achieved new relief, so as to achieve long-term survival, this mode promotes technique to create a new innovative attempt, with an important clinical significance.

Preventive donations of peripheral blood stem cells were conducted 3 months after transplantation in all patients. The infusion was 1 bag of donor peripheral blood stem cells and mononuclear cell count reaches 0.2 × 10^8^/kg. If disease recurrence appeared after transplantation or ETO fusion gene level reaches positive or 10^-6^ levels in more than a logarithmic, we will infuse all the rest of the donor peripheral blood stem cell infusion and all patients show no symptom of GVHD or that of other complications. If patients show symptom of relapse or disease progression after infusion of cryopreserved donor peripheral blood stem cells, and the class II GVHD complications did not appear, according to the patients’ will, we can again collect the peripheral blood stem cell donors with granulocyte colony sampling factor mobilization of 3∼4 days, the blood cell separator was used to collect a peripheral blood stem cells, and the mononuclear cells and CD34^+^ cells was counted. Four bags of cryopreserved peripheral blood stem cells, 1 bag of mononuclear cell meter reached the total number of 0.2 × 10^8^/kg, and the rest of the uniform cryopreserved, four points infusion once a month to patients, GVHD occurred again to the corresponding treatment. The donor peripheral blood stem cell infusion under the purpose of preventing and treating recurrence plays an active role. Different activities are conducted to reduce pretreatment intensity of allogeneic hematopoietic stem cell transplantation therapy for 15 cases of ETO fusion gene positive acute myeloid leukemia patients. All patients were performed with a follow-up observation after the transplantation, the median follow-up duration reached 27.5 months (18∼54 months), and the follow-up lasts till June, 2016. Through statistical survival situation, GVHD occurred in 11 cases, 1 case of death due to complications and recurrence 4 cases died, the other 11 patients remained disease-free survival. 2-year disease-free surial rate was 66.7%, the longest disease-free survival time was up to 54 months and T lymphocyte subsets counts increased significantly before and after the transplantation. 2 cases of death appeared in 6 cases before transplantation for remission, 3 cases of death in 9 cases not ease before transplantation the CPC. The survival curves of the two groups indicate no obvious statistical significance, but our results show that reducing the intensity of pretreatment allogeneic hematopoietic stem cell transplantation in the treatment of ETO(+) delivers curative effect of acute myeloid leukemia. On whether before transplantation patients in remission has nothing to do again, even if relapse status downward hematopoietic stem cell transplantation, there is still a powerful anti-leukemia effect, so as to cure the relapse ETO(+) acute myeloid leukemia. Undoubtedly, the number of cases was small so we cannot reach objective evaluation data from a single center, with more dependence on the number of cases or multicenter study results which were needed in further research.

Above all, the curative effect was good to reduce the intensity of pretreatment of allogeneic hematopoietic stem cell transplantation treat recurrent ETO(+) acute myeloid leukemia curative effect,it gives rise to fewer complications, safety coefficient and high rate of complete response after transplantation. It could cure the relapse ETO(+) in patients with acute myeloid leukemia, can be used as the key technology in clinical extensively developed. The patient bears great tolerance on preprocessing scheme, especially in invalid cases of chemotherapy treatments for high-risk patients. The center is also committed to promoting the work, and innovative preventive after transplantation of donor peripheral blood hematopoietic stem cell infusion, enhancing prevention of leukemia relapse to achieve better effects, with potential clinical application prospects.

## MATERIALS AND METHODS

### Clinical data

15 cases of relapse acute myeloid leukemia (AML) patients in ETO positive had been treated at an army hospital in January 2012 to January 2015. ETO fusion gene positive acute myeloid leukemia was confirmed by cell morphology, histochemistry and molecular genetics. Diagnostic criteria and efficacy judgment are shown in literature. Among these cases, 10 were male and 5 were female, aging from 16 to 48 years old, with the average age of 32.5 years old. The quantity of peripheral blood leukocyte remains > 30 × 10^9^/L during an attack in 6 cases (40%), and chromosome was pure t(8; 21) under observation of 10 cases (66.7%). Additional chromosomal abnormalities were shown in 5 cases(33.3%), c-kit gene mutations in 4 cases (26.7%) and CD56 positive of immune classification in 4 cases (26.7%). All of the patients were adopted with solution under standard dose cytarabine for induction therapy. Afterwards, more than 4∼6 courses consolidation chemotherapy with large dose cytarabine were given to these patients after remission. Median time for suffering from a relapse was 12.2 months (7∼22 months). 5 cases directly undergone hematopoietic stem cell transplantation after relapse while the other 10 adopted FLAG, CAG, IA or large dose Ara-C schemes after relapse, followed which chemotherapy was induced. Six cases underwent allogeneic hematopoietic stem cell transplantation after remission while four wnet through allogeneic hematopoietic stem cell transplantation when failing to remission or suffering from secondary relapse. Median time for confirming to transplant was 13.5 months (8∼32 months). A, B, C, DR, DQ points matched by high resolution molecular biology were carried out for all of donors and recipients. Among them, 9 donors were brothers or sisters of the patients, both of whose HLA matched perfectly with each other. HLA matches of the other 6 donors and recipients were partially mismatched. The treatment in this research was agreed in accordance with both hospital ethics committee and patients’ families. Each viscera organ in all of patients was perfectly functional before transplantation.

### Stem cells mobilization and collection

The communicants of stem cells transplant accepted granulocyte colony stimulating factor (G-CSF) mobilization after physical examination. 2 times on annual basis with dose of 10 ug/kg/d, the treatment consists of subcutaneous injection for 4∼5d. Transplantation method was conducted through peripheral blood stem cell transplantation, collecting peripheral blood stem cell of donors under application of blood cell separator at the fourth and fifth day, in total collection of 2 times. Total number of collected mononuclear cells was more than 5 × 10^8^/kg and CD34^+^ cells more than 2 × 10^6^/kg. Flow cytometry instrument counted number of CD34^+^ cells in the collected material. Preventive infusion for peripheral blood stem cells was conducted in all patients. Peripheral blood stem cell transplantation beyond what would be required were cryopreserved by bag, mononuclear cell count of one bag was 0.2 × 10^8^/kg.

### Preprocessing and GVHD prevention

The whole group of patients were treated with pretreatment solutions of reduced intensity pretreatment (fludarabine plus busulfan, cytarabine and cyclophosphamide). Concrete plans were presented as following. (Fludarabine, 30 mg/m^2^/d × 5 d with -6, -5, -4, -3, -2 days; Cytarabine, 1 g/m^2^/d × 2 d with -6, -5 days; Busulfex, 3.2 mg/Kg/d × 1 d with -4 days; Cyclophosphamide, 40 mg/Kg/d × 2 d with -3, -2 days). Six cases of HLA match half consistency increased application of anti-lymphocyte immune globulin (ATG) during pretreatment, and the concrete remains 2.5 mg/kg with -5∼-1 days. Prevention of graft versus host disease (GVHD) adopted a variety of immune inhibitors in combination, including the ring spore fungus element A, prednisolone, tacrolimus and short-range methotrexate. Blood drug concentration is tested every week. Spore fungus A element concentration in 150∼250 ug/L, and tacrolimus concentration in 5∼15 ng/mL. According to the severity degree of GVHD after transplantation, drugs such as ATG or anti-CD25 and infliximab can be added.

### Complication prevention

All patients before transplantation have to undergo comprehensive physical examination, in order to eliminate potential infection in the areas such as respiratory tract, oral cavity and anus. Patients went into the best grade sterile laminar flow room after medicated bath, indwelling subclavian vein catheter, under the intention of preventing the attack from fungi, cartesian pulmonary cysticercosis, viruses etc. In this process, bowel disinfectant, and infusion immunoglobulin were applied regularly so as to strengthen the composition blood transfusion support treatment. Blood products should be given to patients after irradiation. Fundamentally, broad-spectrum antibiotic treatment was offered after fever empirically, while the secretion culture was performed and ocal infection was found. Hemogram and biochemical are detected every day and blood drug concentration and CMV–DNA detect and fungi G test are performed every week. When neutrophils reached over 0.5 × 10^9^/L for granulocyte implants, platelet came up to over 20 × 10^9^/L for consecutive 3 days without transfusion for platelet implant after transplantation.

### Disease surveillance

All patients were performed disease surveillance after transplantation, including bone marrow morphology inspection at 1 month after transplantation, and quantitative analysis of ETO fusion genes at the same time. In addition, flow cytometry method is adopted for dynamic quantitative detection of residual leukemia immune. ETO fusion gene quantitative positive needed to undergo dynamic monitor every month, with performance of implant test on +30 day, +60 day, +90 day after transplantation and monitor of sex chromosomes by fluorescence *in situ* hybridization technology in case of gender of donor and recipients was different, while assessing implant state for checking chimeric by STR (short tandem repeat polymorphism) polymorphisms method in case of gender was same. We evaluated disease states of all patients after transplantation, and counted disease-free survival.

### Method of chimerism testing

**Figure 8:**
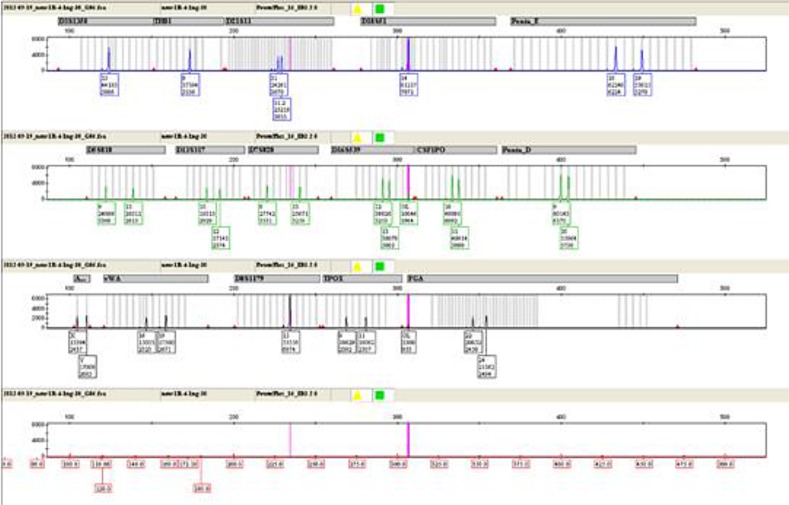
The STR method by three-color fluorescence testing

**Figure 9:**
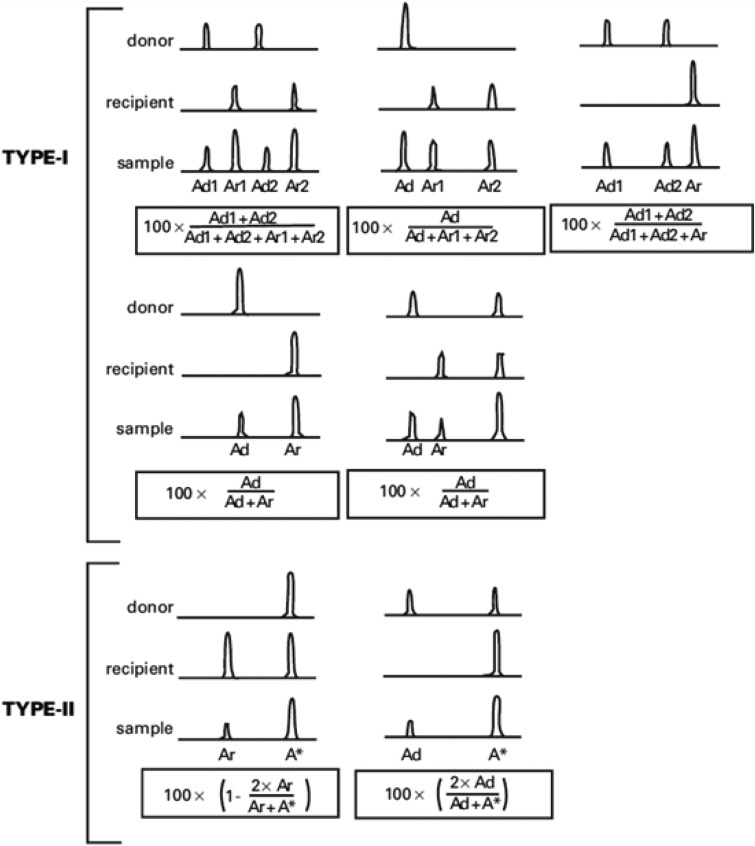
The computing method of donor cell chimerism

**Figure 10:**
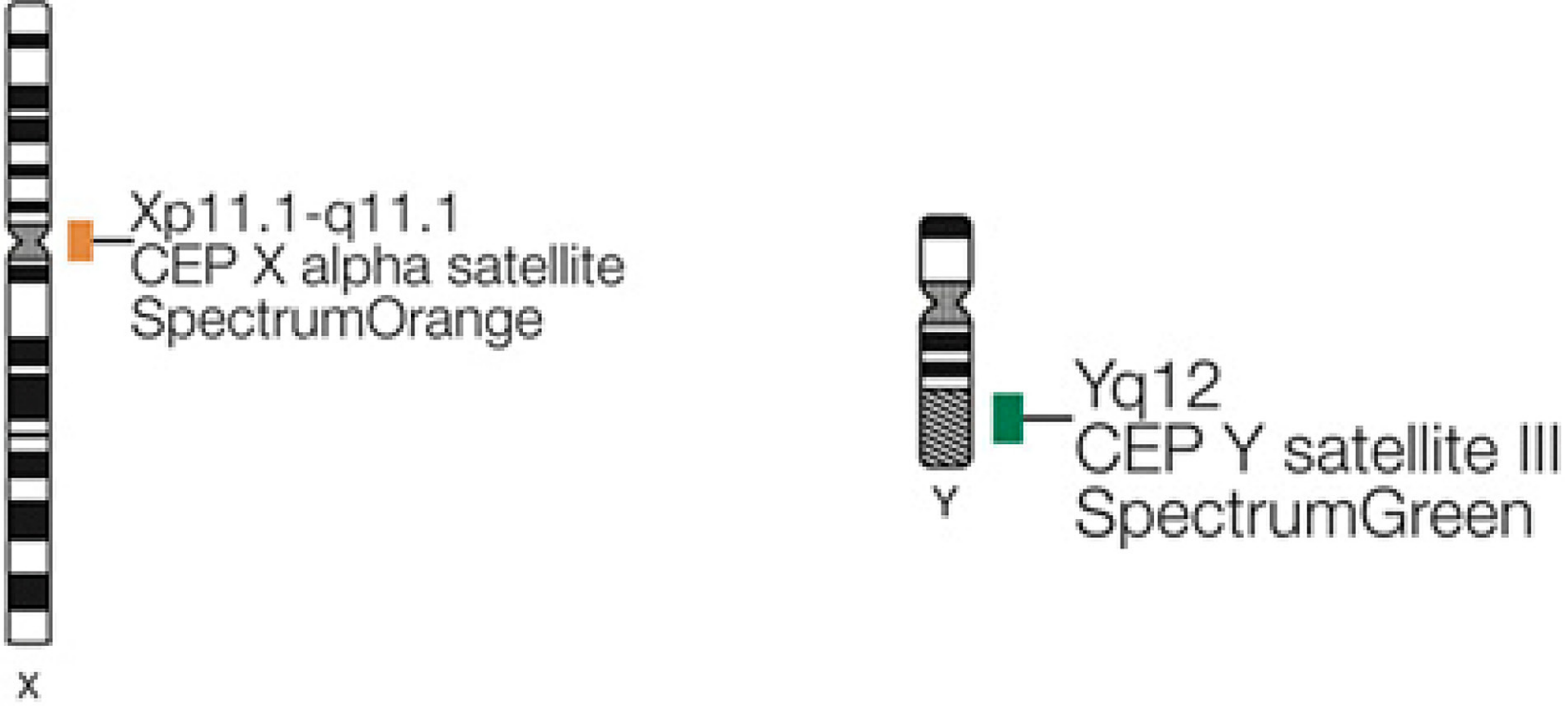
The probe map used for chimerism testing by FISH

**Figure 11:**
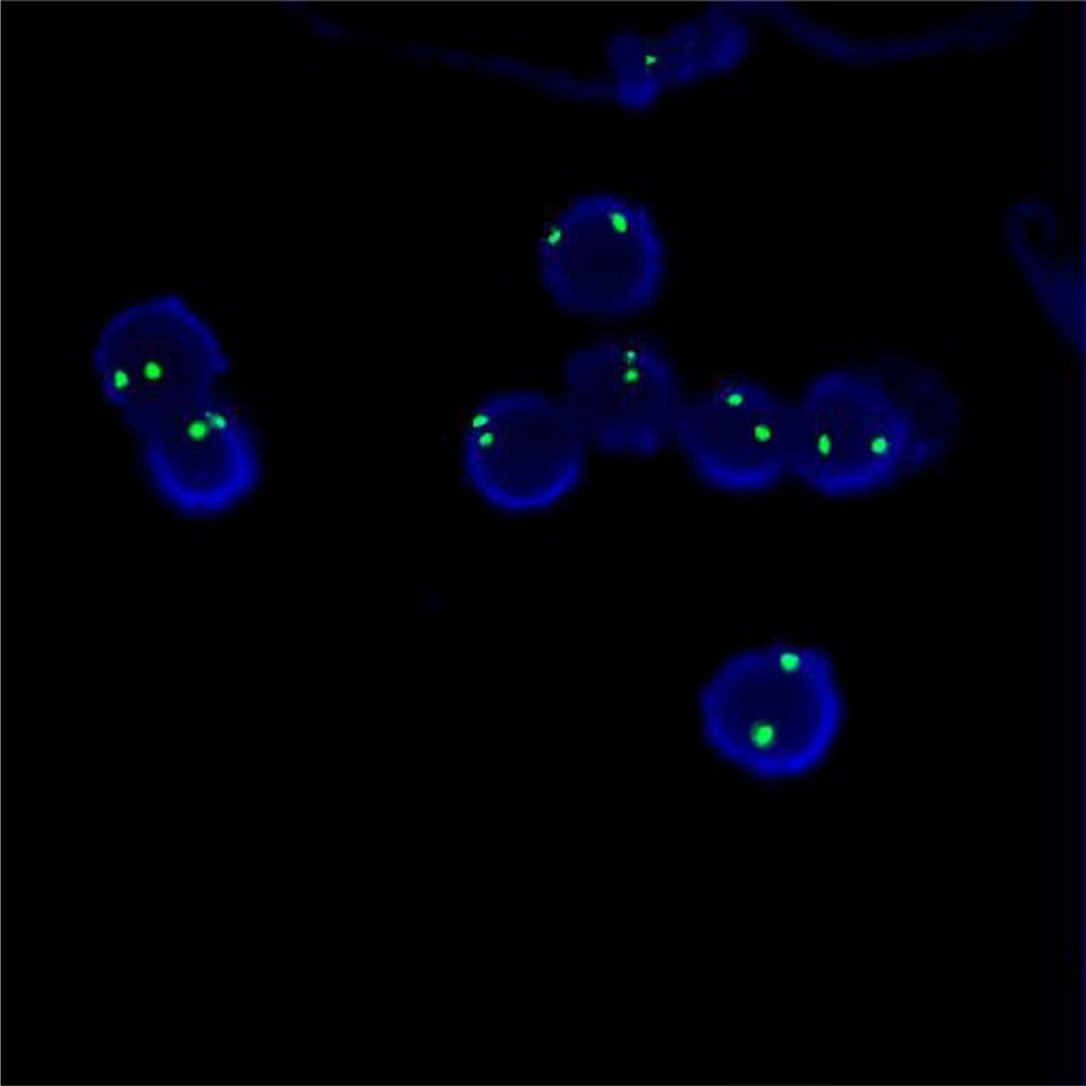
The diagram of the outcome named nuc ish (CEPX) × 2 [495/500]

**Figure 12:**
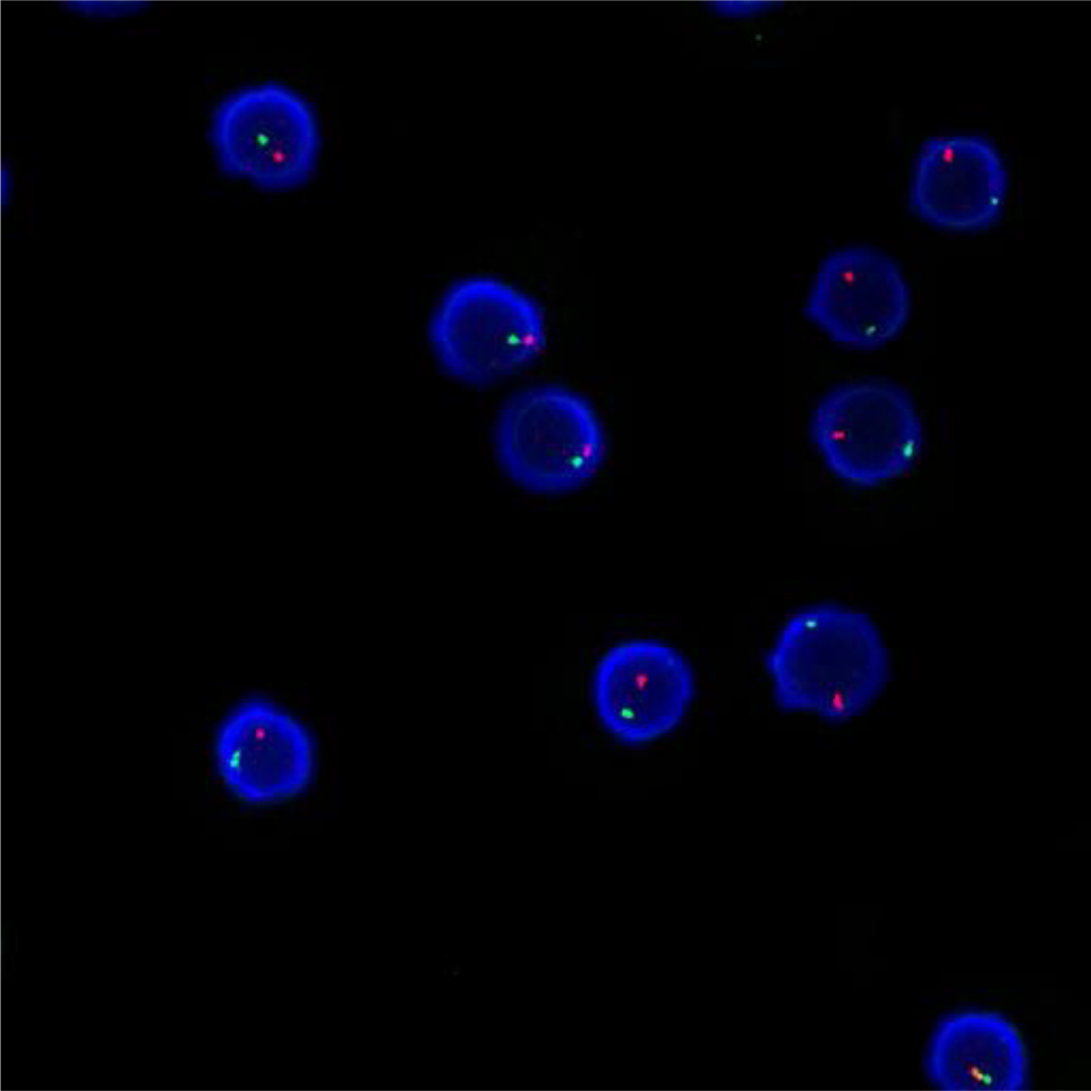
The diagram of the outcome named nuc ish (CEPX, CEPY) [495/500]

Applying the STR technology, polymerase chain reaction(PCR) carries out multiplex amplification of 16 gene loca (namely 15 STR loca and 1 amelogenin locus) and tests by three-color fluorescence (see Figure 8). The design focused on amplimers of these loca and DNA amplification of the same scale was conducted by PCR. Different numbers of replications of repetitive sequences within the amplified region led to typing differences of allele. After electrophoretic separation, different genotypes can be distinguished by fluorescence detection. Before transplantation, peripheral blood or bone marrow of donors and recipients were sampled respectively to identify their STR loca. According to STR loca maps of donors and recipients before transplantation, proportions of cells from donors and recipients, namely donor cell chimerism, can be obtained by calculation (see Figure 9). Fluorescently-labeledDNA probes were used for fluorescence *in situ* hybridization (FISH). By the complementarity between probes and DNA base pairs of samples, after hybridization of probes and DNA of samples, outcomes were obtained to examine chromosomal or genetic abnormality in cells and tissue samples in accordance with fluorescence signals detected with fluorescence microscope. The name of probe used in this study: CEPX SpectrumOrange/Y SpectrumGreen DNA Probe Kit(Model: 7J20–50), probe map (see Figure 10). According to ISCN2013, CEPX/Y was named nuc ish (CEPX) × 2 [495/500], indicating that gene CEPX of 495 out of 500 interphase nuclei had two copies (see Figure 11). CEPX/Y was named nuc ish (CEPX, CEPY) [495/500], showing that 495 out of 500 interphase nuclei had one copy of CEPX and CEPY (see Figure 12).

### Follow-up

All patients were followed-up immediately after transplantation. The median follow-up time was 27.5 months (18∼54 months), and the end of the follow-up time was in June, 2014. Hematopoietic recovery and complications of all patients were analyzed after transplantation, and census mortality, complications, relapse and disease-free survival related treatment of all patients were conducted. Under the assistance of SPSS 16 statistical software for statistical processing and Kaplan Meier method, survival analysis was able to be conducted.

## References

[1] Bots M, Verbrugge I, Martin BP, Salmon JM, Ghisi M, Baker A, Stanley K, Shortt J, Ossenkoppele GJ, Zuber J, Rappaport AR, Atadja P, Lowe SW (2014). Differentiation therapy for the treatment of t(8;21) acute myeloid leukemia using histone deacetylase inhibitors. Blood.

[2] Breig O, Bras S, Martinez Soria N, Osman D, Heidenreich O, Haenlin M, Waltzer L (2014). Pontin is a critical regulator for AML1-ETO-induced leukemia. Leukemia.

[3] Guo Z, Gao HY, Zhang TY, Liu XD, Yang K, Lou JX, He XP, Zhang Y, Chen P, Chen HR (2016). Analysis of allogeneic hematopoietic stem cell transplantation with high-dose cyclophosphamide-induced immune tolerance for severe aplastic anemia. Int J Hematol.

[4] Xuan L, Fan Z, Zhang Y, Zhou H, Huang F, Dai M, Nie D, Lin D, Xu N, Guo X, Jiang Q, Sun J, Xiao Y (2016). Sequential intensified conditioning followed by prophylactic DLI could reduce relapse of refractory acute leukemia after allo-HSCT. Oncotarget.

[5] Park S, Kim K, Jang JH, Kim SJ, Kim WS, Jung CW (2016). Blood concentration of cyclosporine during early post-transplant period may have influence on the occurrence of chronic graft versus host disease in patients who received allogeneic hematopoietic stem cell transplantation. Oncotarget.

[6] Arora R, Sawney S, Saluja D (2016). Potential Therapeutic Approaches for the Treatment of Acute Myeloid Leukemia with AML1-ETO Translocation. Curr Cancer Drug Targets.

[7] Li Y, Wang H, Wang X, Jin W, Tan Y, Fang H, Chen S, Chen Z, Wang K (2016). Genome-wide studies identify a novel interplay between AML1 and AML1/ETO in t(8;21) acute myeloid leukemia. Blood.

[8] Maiques-Diaz A, Chou FS, Wunderlich M, Gómez-López G, Jacinto FV, Rodriguez-Perales S, Larrayoz MJ, Calasanz MJ, Mulloy JC, Cigudosa JC, Alvarez S (2012). Chromatin modifications induced by the AML1-ETO fusion protein reversibly silence its genomic targets through AML1 and Sp1 binding motifs. Leukemia.

[9] Jin J, Li Y, Wang Y, Wang P, Wang Y (2014). SCF/C-KIT signaling modulates tryptase expression in acute myeloid leukemia cells. Int J Hematol.

[10] Park SH, Chi HS, Min SK, Park BG, Jang S, Park CJ (2011). Prognostic impact of c-KIT mutations in core binding factor acute myeloid leukemia. Leuk Res.

[11] Wang CL, Ding BJ, Jiang L, Yin CX, Zhong QX, Yu GP, Li XD, Meng FY (2015). Increased expression of amyloid precursor protein promotes proliferation and migration of AML1/ETO-positive leukemia cells and be inhibited by panobinostat. Neoplasma.

[12] Goyama S, Schibler J, Gasilina A, Shrestha M, Lin S, Link KA, Chen J, Whitman SP, Bloomfield CD, Nicolet D, Assi SA, Ptasinska A, Heidenreich O (2016). UBASH3B/Sts-1-CBL axis regulates myeloid proliferation in human preleukemia induced by AML1-ETO. Leukemia.

[13] Torgersen ML, Engedal N, Bøe SO, Hokland P, Simonsen A (2013). Targeting autophagy potentiates the apoptotic effect of histone deacetylase inhibitors in t(8;21) AML cells. Blood.

[14] Bernardo ME, Piras E, Vacca A, Giorgiani G, Zecca M, Bertaina A, Pagliara D, Contoli B, Pinto RM, Caocci G, Mastronuzzi A, La Nasa G, Locatelli F (2012). Allogeneic hematopoietic stem cell transplantation in thalassemia major: results of a reduced-toxicity conditioning regimen based on the use of treosulfan. Blood.

[15] Gao XN, Lin J, Ning QY, Gao L, Yao YS, Zhou JH, Li YH, Wang LL, Yu L (2013). A histone acetyltransferase p300 inhibitor C646 induces cell cycle arrest and apoptosis selectively in AML1-ETO-positive AML cells. PLoS One.

[16] Ueyama J, Kure A, Okuno K, Sano H, Tamoto N, Kanzaki S (2012). [Treatment with a tyrosine-kinase inhibitor of for c-KIT mutation and AML1-ETO double positive refractory acute myeloid leukemia]. [Article in Japanese]. Rinsho Ketsueki.

[17] Rulina AV, Spirin PV, Prassolov VS (2010). Activated leukemic oncogenes AML1-ETO and c-kit: role in development of acute myeloid leukemia and current approaches for their inhibition. Biochemistry (Mosc).

[18] Zhang L, Li Q, Li W, Liu B, Wang Y, Lin D, Zhou C, Li C, Wang J, Mi Y (2013). Monitoring of minimal residual disease in acute myeloid leukemia with t(8;21)(q22;q22). Int J Hematol.

[19] Zhou GS, Hu Z, Fang HT, Zhang FX, Pan XF, Chen XQ, Hu AM, Xu L, Zhou GB (2011). Biologic activity of triptolide in t(8;21) acute myeloid leukemia cells. Leuk Res.

[20] DeKelver RC, Yan M, Ahn EY, Shia WJ, Speck NA, Zhang DE (2013). Attenuation of AML1-ETO cellular dysregulation correlates with increased leukemogenic potential. Blood.

[21] Gustafson SA, Lin P, Chen SS, Chen L, Abruzzo LV, Luthra R, Medeiros LJ, Wang SA (2009). Therapy-related acute myeloid leukemia with t(8;21) (q22;q22) shares many features with de novo acute myeloid leukemia with t(8;21)(q22;q22) but does not have a favorable outcome. Am J Clin Pathol.

[22] Middeke JM, Fang M, Cornelissen JJ, Mohr B, Appelbaum FR, Stadler M, Sanz J, Baurmann H, Bug G, Schäfer-Eckart K, Hegenbart U, Bochtler T, Röllig C (2014). Outcome of patients with abnl(17p) acute myeloid leukemia after allogeneic hematopoietic stem cell transplantation. Blood.

[23] Stuardo M, Nicovani S, Javed A, Gutierrez S (2013). Breakpoint regions of ETO gene involved in (8;21) leukemic translocations are enriched in acetylated histone H3. J Cell Biochem.

[24] Winkler SH, Barta S, Kehl V, Schröter C, Wagner F, Grifka J, Springorum HR, Craiovan B (2016). Perioperative blood loss and gastrointestinal tolerability of etoricoxib and diclofenac in total hip arthroplasty (ETO-DIC study): a single-center, prospective double-blinded randomized controlled trial. Curr Med Res Opin.

[25] Forster VJ, Nahari MH, Martinez-Soria N, Bradburn AK, Ptasinska A, Assi SA, Fordham SE, McNeil H, Bonifer C, Heidenreich O, Allan JM (2016). The leukemia-associated RUNX1/ETO oncoprotein confers a mutator phenotype. Leukemia.

[26] Rondelli D, Goldberg JD, Isola L, Price LS, Shore TB, Boyer M, Bacigalupo A, Rambaldi A, Scarano M, Klisovic RB, Gupta V, Andreasson B, Mascarenhas J (2014). MPD-RC 101 prospective study of reduced-intensity allogeneic hematopoietic stem cell transplantation in patients with myelofibrosis. Blood.

[27] Punatar S, Gupta A, Gawande J, Bagal B, Mathew L, Kannan S, Khattry N (2016). Chronic Graft Versus Host Disease in Acute Leukemia Patients Undergoing Allogeneic Hematopoietic Stem Cell Transplant: Analysis of Risk Factors, Pattern and Long Term Outcome. Indian J Hematol Blood Transfus.

[28] Yoon JH, Lee S, Kim HJ, Jeon YW, Lee SE, Cho BS, Lee DG, Eom KS, Kim YJ, Min CK, Cho SG, Min WS, Lee JW (2016). Impact of cytomegalovirus reactivation on relapse and survival in patients with acute leukemia who received allogeneic hematopoietic stem cell transplantation in first remission. Oncotarget.

[29] Rio B, Chevret S, Vigouroux S, Chevallier P, Fürst S, Sirvent A, Bay JO, Socié G, Ceballos P, Huynh A, Cornillon J, Françoise S, Legrand F (2015). Decreased Nonrelapse Mortality after Unrelated Cord Blood Transplantation for Acute Myeloid Leukemia Using Reduced-Intensity Conditioning: A Prospective Phase II Multicenter Trial. Biol Blood Marrow Transplant.

[30] Jaime-Pérez JC, Villarreal-Villarreal CD, Salazar-Riojas R, Méndez-Ramírez N, Vázquez-Garza E, Gómez-Almaguer D (2015). Increased bacterial infections after transfusion of leukoreduced non-irradiated blood products in recipients of allogeneic stem cell transplants after reduced-intensity conditioning. Biol Blood Marrow Transplant.

