# Correction: Allogeneic hematopoietic stem cell transplantation for relapsed acute myeloid leukemia in ETO positive with reduced-intensity conditioning

**DOI:** 10.18632/oncotarget.26806

**Published:** 2019-03-15

**Authors:** Zhi Guo, Xiao-dong Liu, Hui-ren Chen

**Affiliations:** ^1^ Department of Medical Oncology, National Cancer Center/Cancer Hospital and Shenzhen Hospital, Chinese Academy of Medical Sciences and Peking Union Medical College, Shenzhen, 518116, China; ^2^ Department of Hematology, PLA Army General Hospital, Beijing, 100700, China

**This article has been corrected:** The correct author information, author affiliation and Corresponding author details are given below:

**Zhi Guo^1,2^, Xiao-dong Liu^2^ and Hui-ren Chen^2^**

^1^ Department of Medical Oncology, National Cancer Center/Cancer Hospital and Shenzhen Hospital, Chinese Academy of Medical Sciences and Peking Union Medical College, Shenzhen, 518116, China

^2^ Department of Hematology, PLA Army General Hospital, Beijing, 100700, China

***Correspondence to:*** *Zhi Guo,* ***email:*** guozhi77@126.com

Original article: Oncotarget. 2018; 9:524-538. https://doi.org/10.18632/oncotarget.22612

